# Ushering in the Silver Age of Telehealth: Addressing Telehealth Disparities for Older Adults With Disabilities

**DOI:** 10.1093/geroni/igab046.2180

**Published:** 2021-12-17

**Authors:** Hilary Touchett

**Affiliations:** VA, Houston, Texas, United States

## Abstract

One silver lining of COVID-19 has been the ushering in of ‘the golden age of telehealth’. However, this unplanned rapid conversion to telehealth left many providers and clinics unprepared to address systemic barriers that adversely affect older adults, particularly those with disabilities. Data from the VA Corporate Data Warehouse suggest that the rapid adoption of telehealth in mental health clinics during COVID-19 widened telehealth utilization disparities for older Veterans (65+) with disabilities. With 4.5 million Veterans 55+ who have at least one disability more attention to addressing this widening gap is needed. For those with hearing, vision, and complex mobility impairments, there are unique challenges to initiating telehealth services. Dr. Touchett will present preliminary findings while discussing ethical and contextual considerations when using telehealth with older Veterans who have disabilities, while discussing ways to facilitate robust clinical encounters for this population.

